# Developing Self-Management Application of Fall Prevention Among Older Adults: A Content and Usability Evaluation

**DOI:** 10.3389/fdgth.2020.00011

**Published:** 2020-09-02

**Authors:** Keren Mazuz, Seema Biswas, Uri Lindner

**Affiliations:** ^1^Management of Service Organizations M.A., Hadassah Academic College, Jerusalem, Israel; ^2^Galilee Medical Center, Nahariya, Israel; ^3^Kaplan Medical Center, Rehovot, Israel

**Keywords:** aging, care services, open innovation, fall prevention, mHealth, self-management App, usability

## Abstract

This paper presents a research and development project for studying aging and technology in fall prevention. Falls are an important global health problem in an aging global population. Up to 50% of serious falls may be fatal. Falls result from the cumulative effects of cognitive, musculoskeletal and sensory decline on postural control and substantially affect the activities of daily living, leading to a lower quality of life and physical injury. A near-fall, misstep and a prior fall are established risk factors for a more serious fall. The fear of falling may reduce physical activity and further predispose to falling. However, limitations in the reporting and documentation of fall events create “silent events”—events that are neither documented nor acted upon. An “Age-Techcare” Application (App) was designed using open innovation methods with local older adult populations and health care professionals through a mixed-methodology approach. The App comprised a digital diary for the self-reporting of fall events and an exercise video to strengthen balance as a fall-prevention intervention. The older adults recorded four fall events: a near-fall, the fear of falling, a fall, or no-fall. Prompts to watch the video and the number of times the video was watched were also recorded on the App. Reports retrieved from the App were analyzed after a 10-week pilot study among older adults accessing the App on their smartphones (*n* = 28) and through their smartTV (*n* = 23). All participants used the App to self-report fall events. Near-falls were the most frequently reported fall event among both smartphone and smartTV groups. The scale of silent falls (including a fear of falling and near falls) is greater than anticipated (according to prevailing literature) and significant, especially among the older cohort of participants who had previously experienced falls and are living alone. The exercise video was regularly accessed within a self-report–fall-prevention feedback loop. Watching a preventive exercise video clip as a preventive intervention is positively associated with self-reporting of all events. We have shown that the utility and effectiveness of an App in the self-management of fall events to raise self-awareness, document risk and prompt preventive action. As we address the health needs of an aging global population, Apps such as this will need to be further developed and interface with health and social care services. The facility for older adults to negotiate ideas and practices of risk and safety—the hallmark of the aging-in-place and healthy aging discourse—is important to them in their acceptance of dynamic and diverse technology.

## Introduction

This paper presents a research and development project for studying aging and technology of fall prevention. Falls are a common and dangerous geriatric syndrome among older individuals (1) and a major cause of morbidity and mortality (2). Resulting from the cumulative effects of cognitive, musculoskeletal and sensory decline on postural control in the activities of daily living, falls constitute an important antecedent of restrictions in daily activities, a lower quality of life and physical injury (3, 4). According to the World Health Organization (5), an estimated 646,000 fatal falls occur every year, making this the second leading cause of unintentional injury-related death in all regions of the world^1^. Death rates are highest among adults over the age of 60 years. These rates are expected to rise with population growth and aging. Medical costs resulting from falls in the USA in 2015 totaled more than $50 billion. Medicare and Medicaid shouldered 75% of these costs (6). Falls, thus, represent a growing global health and economic problem; and, fall prediction and prevention have important roles to play in health promotion.

A fall happens when an individual unintentionally comes to rest on a lower surface. A misstep or “near-fall” is a trip, slip, or other loss of balance in which recovery prevents a fall (7). One of the strongest established risk factors for a fall is a history of a prior fall (8, 9)^2^. Individuals with a history of a greater number of recent falls have a higher risk of a future injurious fall (10). Accurate identification of a fall event (a fall or near-fall) is, therefore, critical to long-term risk stratification (11, 12). However, the reporting of fall events, especially of fall events such as a near-fall or the fear of falling has been shown to be problematic because a detailed description and accurate classification of the nature of each fall require systematic documentation of every fall event: the type of each fall; frequency of falls; and, injury outcomes in each case. Davalos-Bichara et al. (13) suggest that a near-fall, important in predicting a future fall, is not uniformly described in existing fall classification systems (13–16). Specific definition criteria for falls are not typically offered in fall screening and reporting tools used in clinical inpatient and outpatient settings (17); and, the clinical and functional significance of each fall event is not captured by most current definitions (18, 19). Further, patients and clinicians define falls differently.

Fear of falling is another key factor within the fall domain (20) and in its interrelations with falls as a predictor, outcome, or both in the vicious cycle of falls, fear of falling and functional decline (21). This “spiraling risk” means that experiencing any of these phenomena generates the risk of developing the other. However, the fear of falling is seldom recognized or reported by health care professionals.

These limitations in the reporting and documentation of fall events create “silent events”—events that are neither documented nor acted upon, and, as a result, are overlooked, precluding the accurate calculation of risk and effective prevention of falls.

Efforts have been made to consolidate the disparate definitions of fall events. Davalos-Bichara et al. (13) developed and validated the Hopkins Falls Grading Scale (HFGS) supported by illustrations intended to standardize the reporting of near-fall and fall events based on their impact on the patient, and to enhance the accuracy of falls-reporting systems in clinical and research settings. The Falls Efficacy Scale-International (FES-I) registers an individual's level of concern about falling while undertaking 16 social and physical activities inside and outside the home (even if the individual does not actually undertake every activity) (22). Srygley et al. (7) encouraged the self-report of missteps (near-falls) as well as actual falls on a daily calendar mailed to his research team in prepaid and preaddressed envelopes. Subjects were instructed to keep the calendar in a convenient place and to record the number of falls and missteps that occurred after every fall event or at the end of each day. They concluded that self-report of missteps “may enhance our ability to discriminate among older adults with different levels of fall risk and allow for the identification of high-risk individuals before they have a first fall. Thus, this measure may have advantages over the more traditional reliance on the self-report of falls alone” (p. 791).

Mobile phones have been successfully used in several areas of healthcare (mHealth) and self-management tools. Studies showing the benefits of mobile phone technology in disease management and health monitoring are emerging (23, 24). Glynn et al. (25) report the value of self-monitoring in hypertension management. Morrissey et al. (26) suggest that digital health interventions delivered via smartphone applications or connected wireless blood pressure monitors offer a new, scalable, and potentially cost-effective way to improve medication-compliance behaviors. Bengtsson et al. (27) explore the importance of a self-reporting system during periods of labile or uncontrolled blood pressure, especially at the beginning or after a change in a course of medication. Indeed, adapting reporting mechanisms to individual's own smart devices at home is ideal as most falls take place in the home, and responsibility for health awareness and intervention shifts to the individual (28). Thus, investigating how older users feel about and engage with self-management Apps and fall prevention is important.

As the older population grows and effective health care delivery increasingly relies on technology referred to as gerontechnologies (29), older consumers of healthcare technology are of huge market potential (30). Incorporating their opinions into research and design may improve the acceptance and effectiveness of medical technology (31). Little research exists on what older adults really want to understand predictors and barriers to the adoption of technology by older adults (31). A number of factors may prevent elderly users from engaging with technology and healthcare technology, in particular. One of the main factors is ageism, “older adults are often stereotypically described as a homogeneous group that are lagging behind and associated with cognitive decline, frailty and needs” (26, p. 2) which eventually leads to their exclusion from research, development and the design process, so their voices and real needs are not incorporated. Peine and Neven (32) characterized this situation as a “Latourian divide” (p. 2) following the work of Bruno Latour to emphasize the divide between the natural scientists, engineers and designers who are involved in the research, design and production of technologies and the social scientists who work on understanding the intricacies of the life-world of older people, the aging process and how all of this is shaped by other phenomena.

Based on this background, the research questions are: What is the scale of silent fall events in the life of older adults? What is the value of a self-management such tool in fall prevention? And how may such a tool be used in healthy aging?

We considered the following hypotheses:

1. The number of silent falls (such as a fear of falling and near-fall) is greater than falls among the older cohort, especially among those who have had previous falls and are living alone.
2. The design of a feedback loop may increase the value of a self-management App. Thus, watching a preventive exercise video clip as a preventive intervention is positively associated with self-reporting of all events (silent events as well as actual falls).

This research aims to develop and validate a self-management tool of fall events which enables self-reporting and self-implementation of preventive interventions in the form of an online App called “Age-Techcare.” The App was designed to facilitate the documentation of different types of fall events, including silent events, such as a near-fall and the fear of falling among older individuals, and to promote a primary preventive health intervention in the form of an exercise video. As part of the *aging in place* discourse, this App has potential in furthering our understanding of the scale of silent fall events in daily life. Technology for *aging in place* is defined as technology that is developed to support the independence of community-dwelling older adults by alleviating or preventing functional or cognitive impairment by limiting the impact of chronic diseases or by enabling social or physical activity (33).

This research project is based on an open innovation paradigm using agile and mixed-methodology such as quantitative and qualitative data gathered through interviews, focus groups, survey and data retrieval from the App database. Open innovation is a dynamic and continuous process of sharing knowledge, skills and experience within a stakeholder network when developing a product, system or related service. It usually takes place in a multi-stakeholder network with a shared common interest or value through the collaboration to promote continuous iteration of development and testing. This has become standard practice in addressing societal challenges such as healthy aging (30).

As part of the open innovation paradigm, this research was managed and funded by CDI-Negev in collaboration with the Israeli National Insurance Fund of the National Insurance Institute (*Bituach Leumi*)^3^, JDC-Joint (the American Jewish Joint Distribution Committee)^4^, the Israeli Ministry of Health's National Program for Fall Prevention, WizeCare Technologies and Uniper Care Technologies.

The Center for Digital Innovation-Negev (CDI-Negev) living laboratory works with older adults in Beer Sheva, Israel, to promote digital literacy and healthy aging in collaboration with local social, educational, health care and senior citizen advocacy organizations. CDI-Negev develops and tests technological innovations that support healthy aging among senior citizens who receive training in their use in the laboratory, at home and in nearby residential homes. Participants in this research were recruited through the CDI-Negev SeniorTech program.

Uniper Care Technologies^5^ offer an artificial intelligence (AI)-based in-home assistance platform for older adults, producing Android-based set-top boxes that transform any television into a smartTV. In addition to entertainment, the interactive TV platform features a number of holistic services including social engagement, assistance in performing daily activities, and management of medical needs in order to facilitate independent living for older adults in their own homes. WizeCare technologies designed the exercise video which was embedded in the App. WizeCare (https://wizecare.com/) provides all-in-one solutions for tele-rehabilitation physiotherapy. The video comprised static and dynamic balance exercises combined with strength, flexibility, and aerobic exercises.

## Materials and Methods

### Ethical Approval

Ethical approval for all phases of the research was granted by the Ethics Committee of Hadassah Academic College in Jerusalem. All participants received verbal and written information about the research and gave signed written informed consent.

### Inclusion and Exclusion Criteria

To implement an open innovation methodology, all men and women aged 60 years and older, retirees and community-dwelling older adults living in their own homes, Hebrew speaking (as the App was developed in the Hebrew language only), with or without a history of falls and familiar with smartphones and/or smartTV were eligible to take part. Community members with mobility problems were excluded from pilot-testing of the App (as this involved using the exercise video) but some of them were included in interviews and focus group discussions.

None of the participants had previously used an App to manage fall events prior to this study. As this was a novel concept and device, the sampling criteria were designed to capture the older adults' diversity in health, gender, interest in technology, technological innovation and technological literacy. Any or all of these have a potential effect on how a new technological innovation is or is not accepted, how it is used and how it may be modified.

### Research Design

The research and development project comprised three phases and was performed between July 2017 and September 2019. Table 1 shows the three phases in the development and assessment of the App.

**Table 1:** Components of the research and development process of the App.

	**Purpose**	**Number**	**Product**
**PHASE 1: DESIGN AND DEVELOPMENT OF THE APP**			
Research methods	Assessing the participants' knowledge about fall prevention and acceptability of a self-report system	Older individuals (*n* = 10)	Item drafting of the first mock-up
Focus group	Data synthesis	Health care professionals (*n* = 8)	Item drafting of the first mock-up
Focus group	Development of self-report screens of the App	Older individuals (*n* = 12)	A mock-up draft
Focus group	The mock-up validity	Older individuals (*n* = 12)	A prototype
**PHASE 2: TESTING THE APP**			
Survey and guidance meeting	Launching the experiment, registration and guiding the participants	Older individuals (*n* = 51)	Sociodemographic survey and screening for fall events downloading the App from the store and first use on the mobile smartphones and smartTVs
**PHASE 3: ASSESSMENT OF THE APP–USAGE AND ANALYSIS OF REPORTS**			
Automated Information	Analyzing the information accumulated within App reports and on the App system	Older individuals (*n* = 51)	usability
Focus groups (2)	Assessing usability and participants' satisfaction in each group	Group 1 Older individuals using mobile smartphones (*n* = 13) Group 2 Older individuals using smart TVs (*n* = 15)	usability and acceptability
Survey	Assessing usability and participants' satisfaction	Older individuals (*n* = 51)	Correlation of results

#### Phase 1: Design and Development of the App

This phase included the recruitment of participants aged 62 years and over and health care professionals (including doctors, nurses, an employment therapist, social worker and physiotherapist working with the older adults and treating fall events) for focus group input into the design and assessment of a mock-up App. CDI-Negev SeniorTech and Uniper Care smartTV users were invited via email and telephone to join the research process. Those interested were asked to complete a registration form detailing their age and device preference (smartphone or smartTV). Those who registered were then invited to participate in phase 1 focus group discussions and open interviews aimed at item drafting and design. In the focus groups the older adult participants were asked to describe their experience of fall events and their thoughts on the most effective fall-prevention interventions. Discussions focused on the format of the digital diary and the phrasing of self-report questions and responses available for selection from a digital menu. Based on their input, the digital diary comprising seven multiple choice questions with the option to select only one answer to each question (Q&A) and the ability to navigate backwards and forwards through the App, was developed. After building a mock-up, the participants in the focus group interacted with paper drafts of App screens. Based on their feedback, modifications were implemented iteratively until a prototype App was built to operate on smartphone (Android and IOS operating phone systems) and smartTV (Uniper Care Technologies) platforms. Extending the use of this App for smartTV users is crucial in understanding the value of self-management tools among older adults because watching and using TV is a lifelong habit that is deeply rooted in everyday routines as opposed to the new smartphone devices. However, using the smartTV for self-management practices entails learning new practices.

#### Phase 2: Testing the App

This phase involved testing of the App for acceptability and usability in a 10-week pilot study. A total of 51 volunteers were recruited (15 volunteers were Uniper Care smartTV users and 36 were from the CDI-Negev SeniorTech community). Content validity and usability were tested during pilot testing of the App over 10 weeks with the 51 volunteers (*n* = 51, age range = 62–92 years, median age = 74.73) who used the App on their smartphones (*n* = 28) and smartTVs (*n* = 23) according to their preference. All the participants were able to download the App free of charge. Phase 2 began with a group meeting of all participants shown how to download, register and handle the self-report system using their own mobile phones and TVs, how to conduct the exercise training, and how to navigate the App using their smartTV remote control. All participants were asked to complete a sociodemographic survey including their fall history 12 months prior to participation in the study.

Over the 10-week pilot period automated App notifications were received by participants three times a week (on Sunday, Tuesday and Thursday) as reminders to complete their digital diaries and to watch the exercise video. All App users were able to watch and perform these exercises alone at any time. The 10-min video comprised 8 different exercises, each lasting 15–30 s with a trainer demonstrating each technique and counting out exercise repetitions. The App included the facility to play, pause, rewind, replay or forward the exercise video.

#### Phase 3: Date Retrieval and Analysis

In phase 3 the data accumulated in the App, survey and focus group were transcribed, anonymized and stored in a password protected file available only to the researchers for analysis. SPSS (Statistical Package for the Social Sciences) program for *t*-test and Anova associations was used for statistical analysis. Phase 3 concluded with a satisfaction survey and focus group feedback.

## Results

### Phase 1 Results: Components and Design of the App

In phase 1 interviews and focus groups, the participants were asked to describe a fall event they, a relative or friend had experienced in- or outdoors. All the participants' narratives included at least one event of a near-fall or/and fear of falling, whether in the shower at home or outdoors on stairs or a bus. Moreover, all the participants concluded that they do not routinely report these events to their caregivers or physicians. They gave three reasons for this: firstly, they do not want to be a burden and to add more issues and problems to bother their caregivers so they preferred to report only an actual fall event which caused them a major injury for which they required immediate treatment; secondly, they feel ashamed to share the details of every fall event and moreover they are afraid of losing their independence and control if all their fall events become known and their vulnerability exposed; and finally, every participant perceived that no interventions existed after report of a silent fall such as the fear of falling or near-fall, thus, no value was perceived in reporting a silent event. In addition, reporting a fall to their physician entailed referral to physiotherapy which they preferred not to attend as this was at additional cost and trouble. Thus, silent events are underreported and not acted up on.

The participants described in detail the events following a specific timeline- before and after the event. They described where the fall event happened, when and how it happened, why they thought it had happened and what they had done just before and after the event.

Based on these narratives, the questions in the mock-up (and subsequent App) were constructed.

The flowchart in Table 2 shows the navigation design through the questions in the App. Screens 1–6 represent the digital diary Q&A and screens 7–8 represent the preventive intervention. Participants were at pains to phrase questions and responses in terms they found acceptable. For example, in the first question on the home screen “what happened to you?” they suggested four answers:

- I fell
- I nearly fell
- I fear falling
- I had a good day (meaning “no fall”).

**Table 2:** Flow chart illustrating the logic process of the App.

	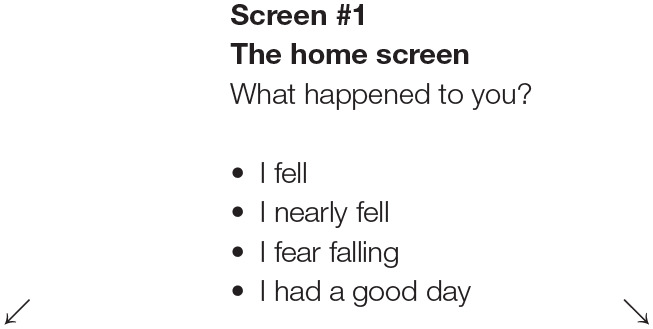	
**Answer options:** • I fell • I nearly fell • I fear falling **Move to screen #2**		**Answer options:** • I had a good day **Move to screen#7**
**Screen#2** When did it happen? 1. Day 2. Night **Move to screen #3**		
**Screen#3** Where did it happen? • Indoors (move to screen #3A) • Outdoors (move to screen #3B)		
**Screen #3A** If the event happened outdoors, where did it happen? 1. On the bus 2. In a street 3. In a building 4. On the stairs **Move to screen#4**	**Screen #3B** If the event happened indoors, where did it happen? • In the kitchen • In the shower/toilet • In the bedroom • On the stairs **Move to screen#4**	
**Screen#4** What do you think caused the event? 1. Slippery surface 2. Tripped 3. Dizziness 4. Unsafe behavior 5. I don't know **Move to screen #5**		
**Screen#5** What had you just done before the event? 1. Got up from seat 2. Taken medication 3. Used the stairs 4. I don't know **Move to screen #6**		
**Screen#6** Did you seek medical treatment after the event? 1. Yes 2. No **Move to screen #7**		
	**Screen#7** Thank you for joining us, let's begin a balance exercise. This series of activities can help reduce the risk of a fall. The lesson will take up to 10 min so wear comfortable closed footwear and choose a suitable place to do your exercises. If you feel weak or dizzy stop the activity. Enjoy! **Let's begin**	
	**Screen#8** How was it? 1. Like 2. Dislike	


The phrase “I had a good day” was used instead of “no fall” at the insistence of focus group participants who perceived this a more positive description and a sharper contrast to the other options. Another phrase the participants offered was “I don't know” instead of “I don't remember” so that there was no ageist implication of memory loss. The participants suggested that automated notifications to complete the diary be sent three times per week as a helpful friendly reminder and a habit-forming refrain at an hour of their own choice. After downloading the App, the registration process on the App was as follows:

1. Login and registration of personal details including name, age, gender
2. Password selection to complete login
3. Customization of the hour of notifications.

Co-creating the App with the participants contributed to the understanding of the meanings of fall events in the everyday life of older adults and, as a result, shaped the design logic.

The design premise was to create a feedback loop between the self-reports (“what happened to me?”) and the intervention (“what can I do?”) with one action linking to and driving the other, so self-reports became the driver to increase awareness and motivate the implementation of preventive interventions. The App delivers, at once and in one click, both the self-reporting diary and the prevention intervention—downloading and performing fall-prevention exercises.

### Phase 2 Results: the Scale of Silent Fall Events

A total of 51 volunteers were registered for testing pilot of the App. Most (n = 45) of them were new to the project and had not participated in phase 1. As presented in Table 3, according to the baseline survey, the age range was from 62 to 92 years. The median age of mobile smartphone users was 72 years, and of smartTV users 78 years. More than half of the smartTV users lived alone.

**Table 3:** Sociodemographic baseline variables of pilot-test participants.

**Participants (*n* = 51)**	**Mobile Smartphone**	**Smart TV users**
	**users**	
Total number	*n* = 28	*n* = 23
**Gender**		
Women	*n* = 20	*n* = 16
Men	*n* = 8	*n* = 7
**Median age** (range)	72 (62–81)	78 (62–92)
**History of falls**		
No fall	*n* = 16 (50%)	*n* = 11 (42%)
One fall	*n* = 9 (32%)	*n* = 11 (42%)
Two or more falls	*n* = 3 (10%)	*n* = 1 (4.3%)
Fear of falling	*n* = 20 (60%)	*n* = 16 (61%)
**Housing**		
Live alone	*n* = 10 (35%)	*n* = 12 (52%)
**Digital literacy**		
Routine smartphone App use	93%	68%
for communication		

In terms of digital literacy, 93% of participants in the smartphone group had used Apps for communication in contrast to 68% of smartTV users. The incidence of prior falls was the same in both groups, as was a 60% fear of falling.

All participants logged into the App three times a week after receiving the automated App notification reminder. Only 10 participants logged in spontaneously at other times. This, they reported, was specifically to watch the exercise video.

As seen in Table 4, a total of 1,210 reports were documented on the App from 51 participants, representing 79% of the total 1,530 notifications that were sent during the 10-week pilot. Additionally, the exercise video was watched a total of 665 times (431 times by the smartTV users). Smartphone users self-reported more often than the smartTV users but smartTV users watched the exercise video more often. Overall, smartTV users reported more silent fall and fall events than the mobile smartphone users and were the older cohort (mean age 78).

**Table 4:** Reports recorded on the App.

**Participants**	**Smartphone**	**Smart TV**	**Total**
	**users (*n* = 28)**	**users (*n* = 23)**	**(*n* = 51)**
**Total number of reports on**	*n* = 709 (81%)	*n* = 501(70%)	*n* = 1,210
**App (*****n*** **=** **1,530) Specific reports**			
“I had a good day” (no falls)	*n* = 648 (91%)	*n* = 386 (77%)	*n* = 1,034
Near fall	*n* = 30 (4%)	*n* = 66 (13%)	*n* = 96
Fear of falling	*n* = 24 (3.3%)	*n* = 27 (5.3%)	*n* = 51
Falls	*n* = 11 (1.5%)	*n* = 18 (3.5%)	*n* = 29
**Response after exercise video**			
Total	*n* = 234	*n* = 431	*n* = 665
Good	*n* = 223	*n* = 332	
Not good	*n* = 9	*n* = 16	
No answer	*n* = 2	*n* = 83	

These results indicate that mobile smartphones were more acceptable for self-reporting while the TV was preferred for watching the exercise video.

In both groups, the event most frequently documented was a “near-fall” (*n* = 96). More than a third (34%) of all documented indoor events happened in the shower or toilet. Half (51%) of the documented outdoor events happened in the street. Half of all users (*n* = 25) answered “I don't know” to the question “what were you doing before the event?.” Almost a third (28%) of these events were preceded by a period of dizziness.

As presented in Table 5, a *t*-test was carried out to analyse the significance of differences between the three groups in their App reports and in relation to dependent variables.

**Table 5:** Statistical analysis in both groups using *t*-test (R_s_).

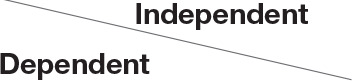	**Fear of falling**	**Near falls**	**Falls**
Prior falls	0.306^*^	0.528^*^	0.815^**^
Age	0.360^*^	0.481^*^	0.124^**^
Living alone	0.100^*^	0.929^**^	0.016^*^

* *p < 0.05.*

** p < 0.01. ^***^ p < 0.001.

Prior falls were significantly and positively associated with more self-reports on the App of a fear of falling and near falls. Participants who did not have a prior fall reported on the App less frequently a fear of falling (M = 6) than those who had fallen once (M = 13) and those who had fallen twice (M = 10).

Age was as an independent predictive factor of silent events. Participants who had fallen once were the oldest (M = 77.35). Age was significantly and positively associated with the fear of falling and near-fall reports on the App. Participants who live alone reported more fear of falling and fall events on the App.

As presented in Table 6, analysis of variance (ANOVA) was carried out to examine the significant mean differences among users who reported “having a good day.”

**Table 6:** Independent variable: “having a good day” (no-falls).

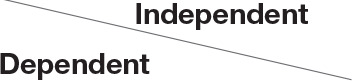	***N***	**M**	**ANOVA test**
No prior falls	22	19.5	–
One fall	18	14.05	–
More than two falls	10	12.4	–
Age	–	–	0.156^**^
Living alone	–	–	0.536^**^

^*^ p < 0.05.

** p < 0.01. ^***^ p < 0.001.

Users who had no prior falls reported more no-fall events on the App (M = 19.5). Among them, no significant differences were found according to age and living alone.

These validate the first hypothesis, that the scope of silent falls such as fear of falling and near-falls is higher than falls among the oldest participants who had previously experienced falls and live alone.

### The Self-Reporting App Feedback Loop

As described in Table 7, a *t*-test was carried out to analyse the significance of differences between the four groups according to their App reports and in relation to the dependent variable (watching the exercise video). A significant positive association was found between the number of times the exercise video was watched and all four of the fall options reported.

**Table 7:** Statistical analysis in both groups using *t*-test (R_s_).

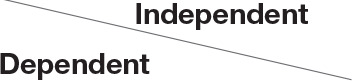	**Fear of falling**	**Near falls**	**Falls**	**No falls**
Video watch	0.163^*^	0.242^*^	0.235^*^	0.517^*^

* p < 0.05. ^**^p < 0.01. ^***^p < 0.001.

This indicates that users completed the feedback loop using all of the four fall options - especially those who reported the most “good days”/no-falls.

The older cohort (smartTV users) watched the video clip 50% more (*n* = 431) than mobile smartphone users (*n* = 231) and reported at least one fall prior to their participation in pilot testing. A significant difference between the smartphone and smartTV users was the association between the number of times the video was watched and the number of fall events as presented in Table 8.

**Table 8:** Statistical analysis between the two groups using *t*-test (R_s_).

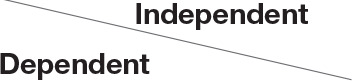	**Fear of falling**	**Near falls**	**Falls**	**No falls**
Video watch smartphone users	−0.319^*^	−0.317^*^	0.159^**^	0.551^*^
Video watch smartTV users	0.377^*^	0.456^*^	0.080^**^	0.665^*^

* *p < 0.05.*

** p < 0.01. ^***^p < 0.001.

A negative and significant association was found among mobile smartphone users between the number of times the exercise video was watched and the number of reports of near-falls and fear of falling. A positive and significant association was found between the number of times the exercise video was watched and the number of “no-fall” reports.

In contrast, among the smartTV users a positive and significant association was found between the number of times the video was watched and the number of near-falls, fear of falling and no-falls. Thus, the group with a prior history of falls watched the video clip most often. In the final focus group, participants agreed that they found the exercises in the video challenging, but they continued to exercise as the preventative measure reassured them and helped them face up to the risk of falling again. Thus, the App was of most value for those most at risk.

Interestingly, in both groups a positive association was found between the number of times the exercise video was watched and the number of “no-fall” reports. The implication is that the more often the video was watched, the more “no-fall” events were reported and vice versa. This potentially strengthens the act of self-reporting in order to motivate the users to undertake preventive exercises, even if the user has not experienced a fall.

This supports the second hypothesis that the design of a feedback loop can increase the value of a self-management App; thus, watching a preventive exercise video clip is positively associated with reports of all fall events (fear of falling, near-falls, falls and no-falls).

Table 9 shows the participants' satisfaction survey results in terms of the utility, effectiveness and acceptance of the App.

**Table 9:** Satisfaction survey results among both smartphone and smartTV users scale is between 1-Strongly disagree, 2-Disagree, 3-Neutral, 4-Agree, 5-Strongly agree.

**I shall continue to follow the fall prevention recommendations in the video**	** 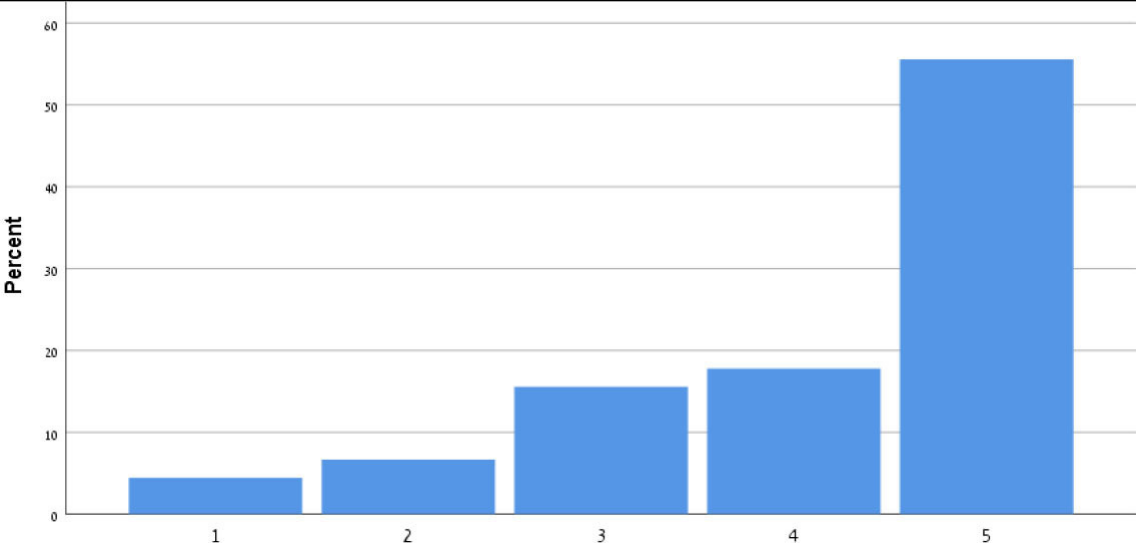 **
I feel safe in reporting personal data in the App	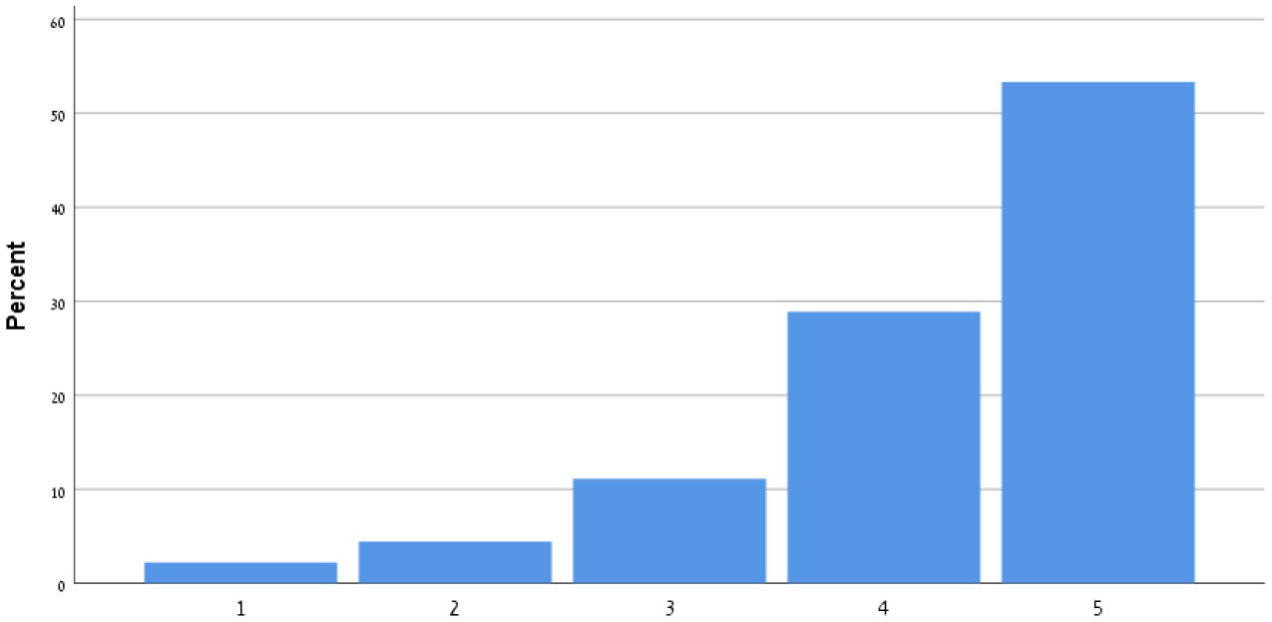
I am satisfied with the App in its current design	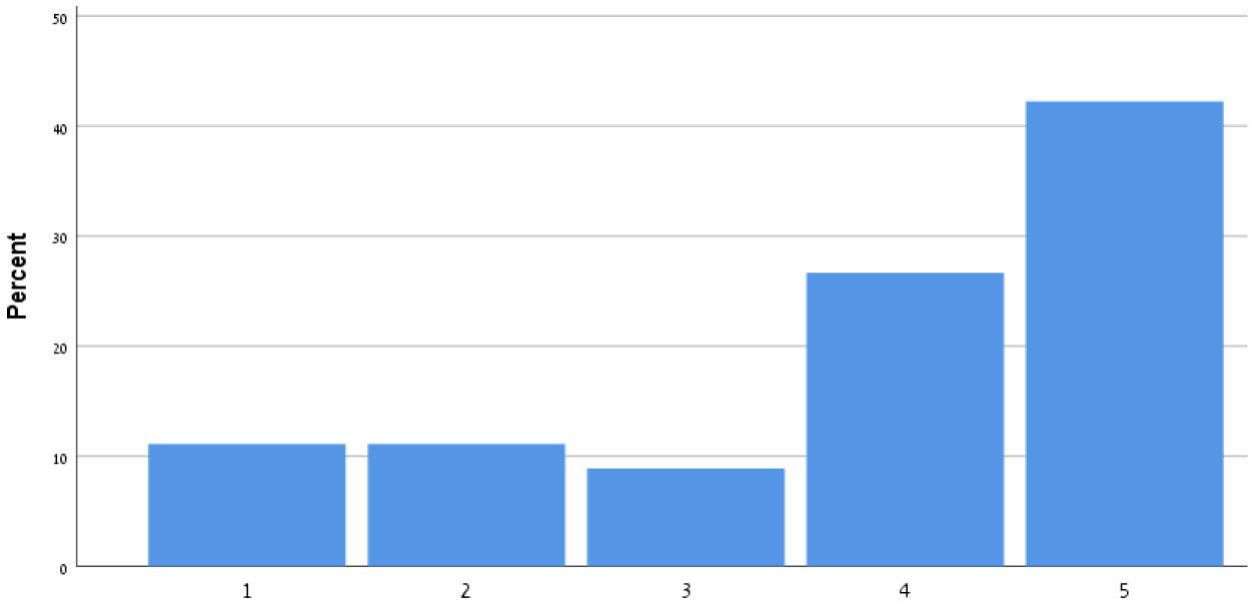
I will recommend that my friends use the App	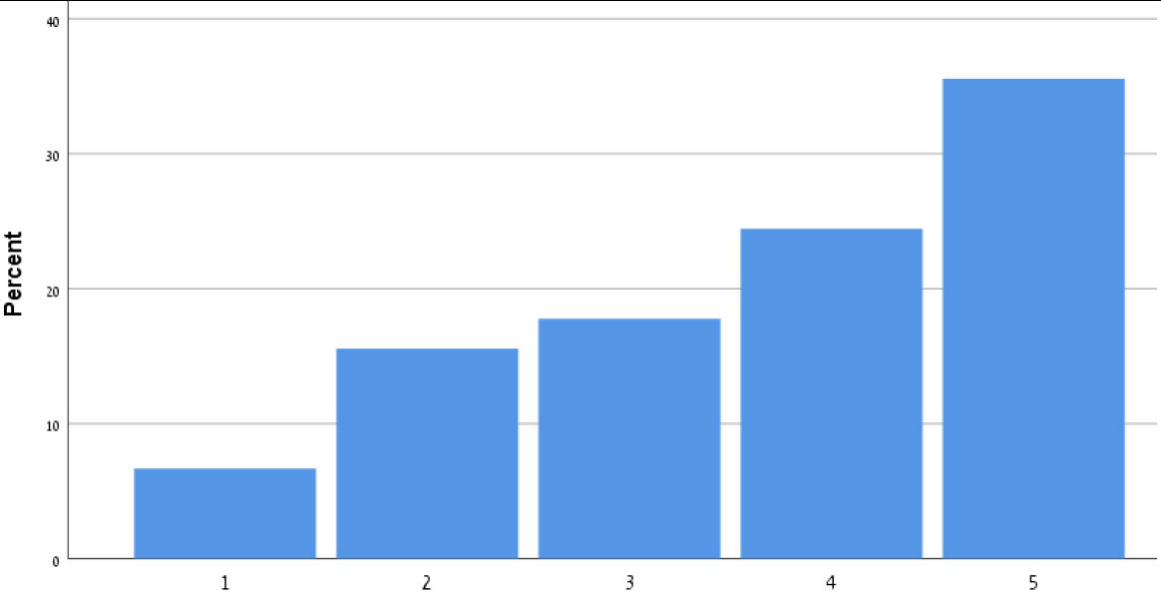
Using the App taught me how to prevent falls	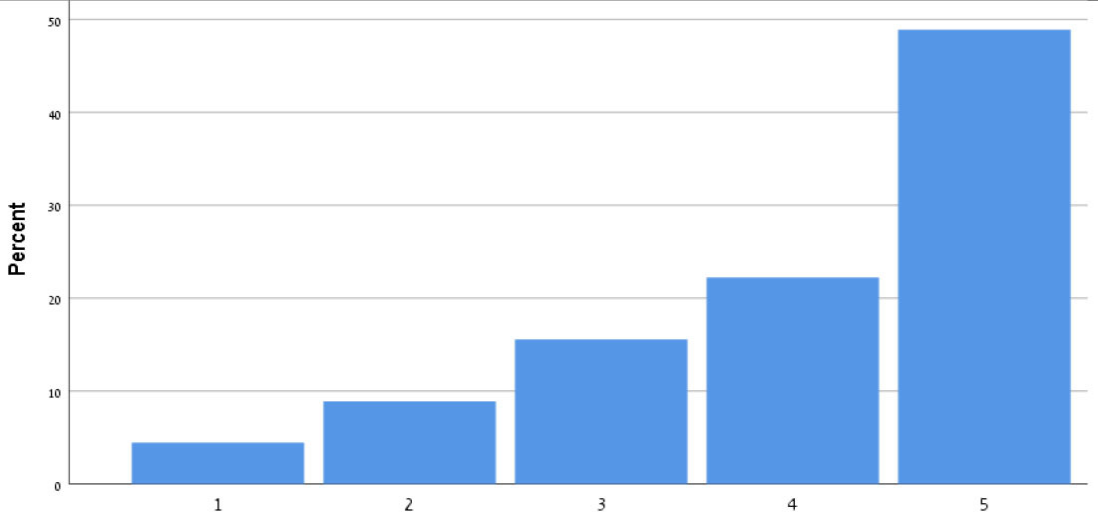
Notification three times a week was appropriate	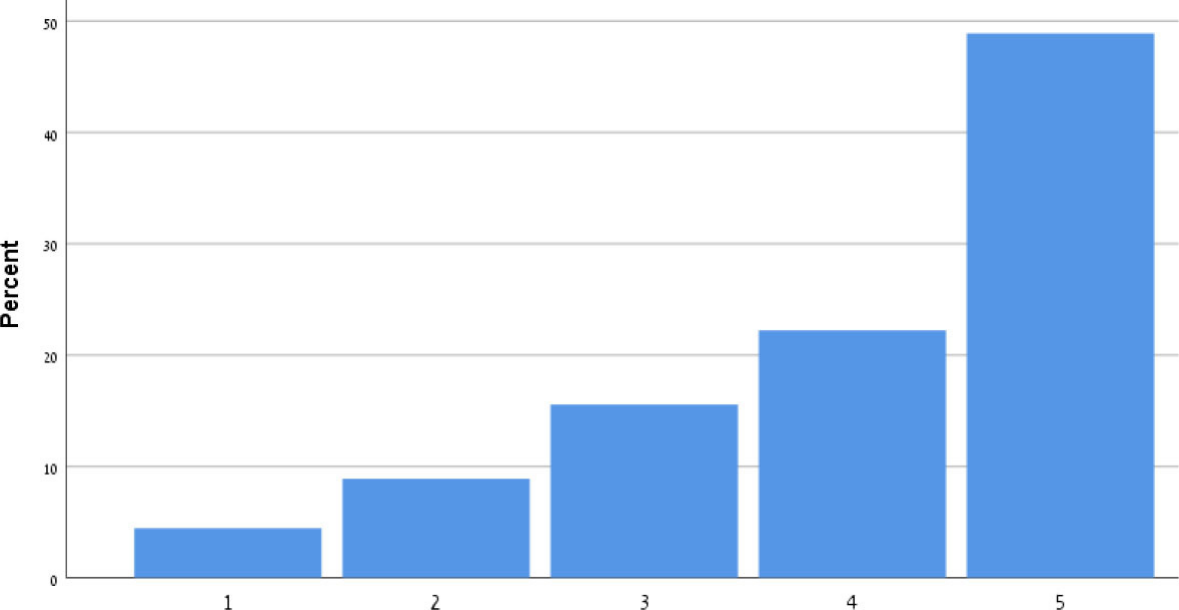
The App is important in the promotion of healthy aging	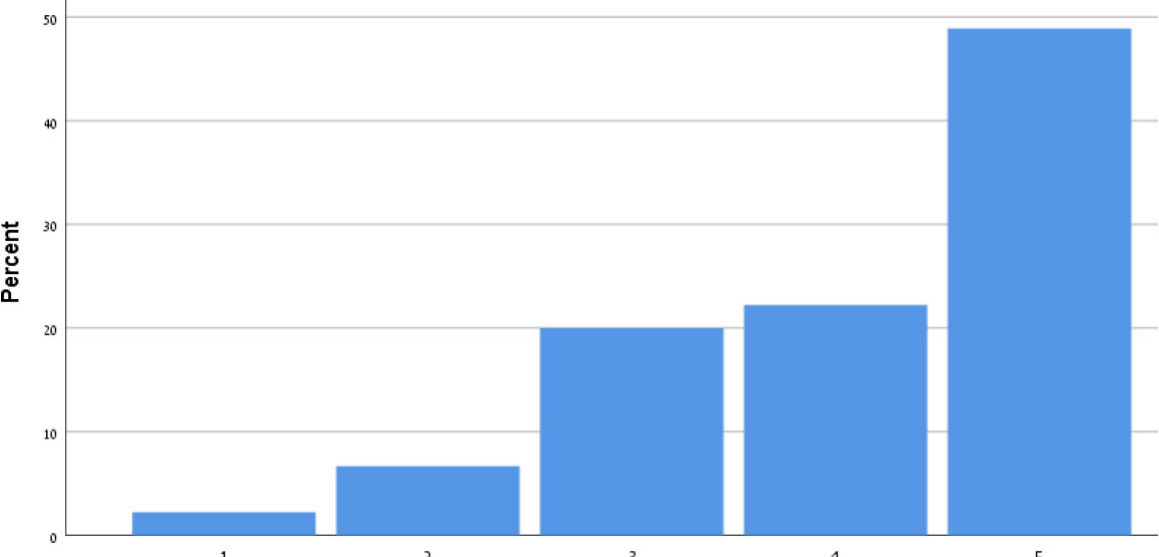
By using the App, I feel I am taking responsibility for my health	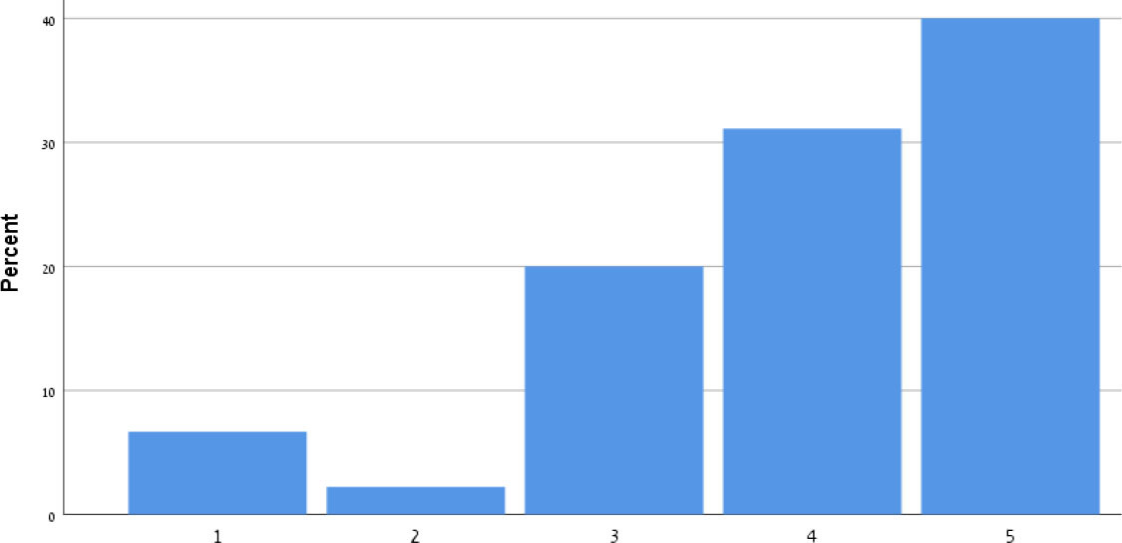
Through the use of the App, I learn that falls can be prevented	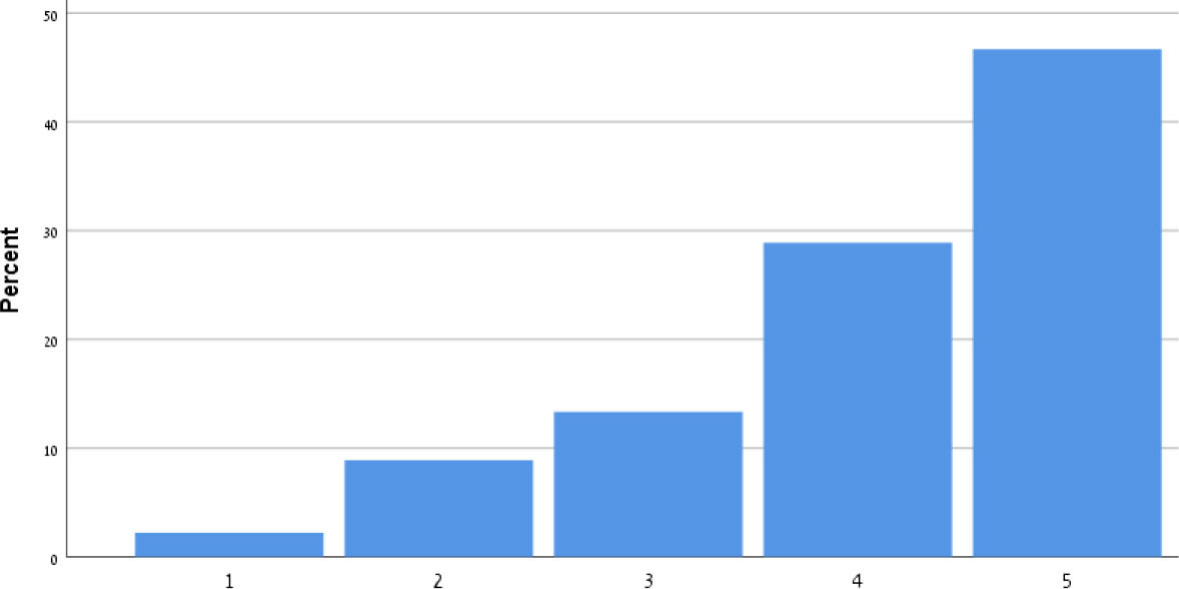
As a result of my participation in this program and using the App, I am taking precautions to prevent a fall	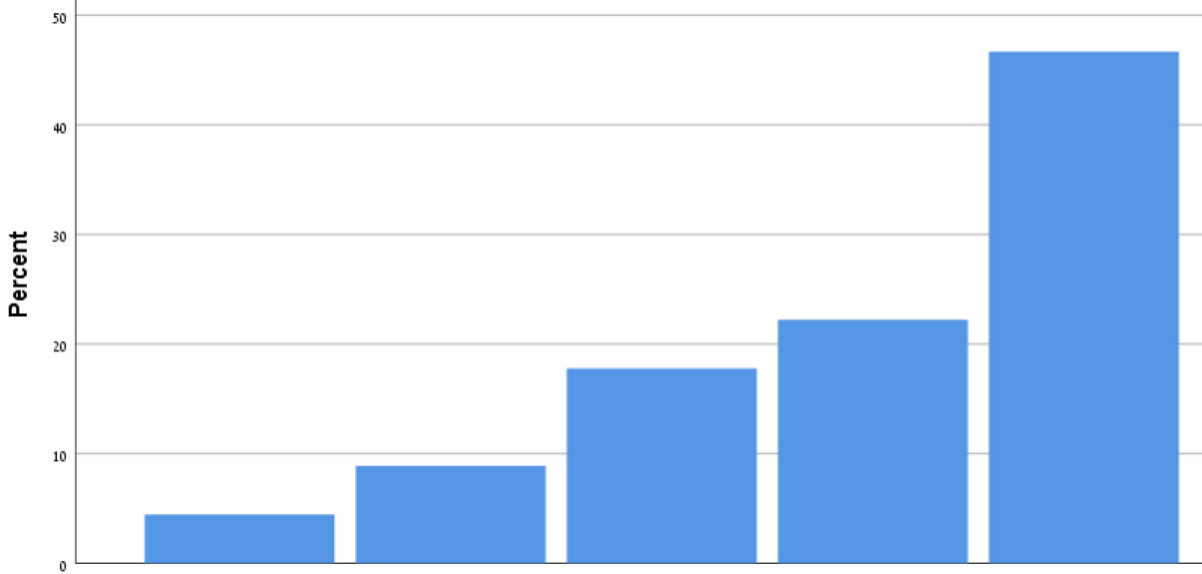

Over 50% of participants among both groups agreed that they would continue to follow the fall prevention recommendations in the video.

When the participants were asked about the meaning of the App in relation to fall events and fall prevention, over 40% of both groups agreed that using the App taught them how to prevent falls and that, through the use of the App, they learned that falls can be prevented. Almost 50% agreed that as a result of their participation in this research and in using the App, they were actively taking precautions to prevent a fall.

More than 50% of both groups of participants agreed that, by using the App, they felt they were exerting responsibility over their own health, and that the App is important in the promotion of healthy aging. Over 50% agreed they would recommend the App to their friends to use.

In terms of usability, more than 50% were satisfied with the App in its current design, with three times weekly notification reminders, and with personal data safety.

## Discussion: How This Self-Management App Constitutes Healthy Aging?

Through this agile and open innovation research with older adults in the participatory design and testing pilot of the “Age-Techcare” App, we found evidence in support of the first hypothesis that the scale of silent falls (a fear of falling and near-falls) is higher than actual reported falls among the oldest participants, especially those who have previously experienced falls and live alone.

The design of a feedback loop did increase the value of a self-management App—supporting the second hypothesis. We found that there were more self-reports than video clips watched, but watching the preventive exercise video clip was positively associated with reports of all events including no-fall reports. In light of our understanding of the scale and increased significance of silent fall events, a self-management tool which enables weekly self-reporting of different types of fall events including “good days”/no-falls while promoting a preventive intervention, is of substantial value as the feedback loop increases awareness while effecting fall prevention. Further, the added value of advocacy and education (“taking responsibility over my own health” and “the App is important in promotion of healthy aging”) potentially empowers and builds confidence. As silent events are harbingers of serious falls (up to 50% of which are fatal), reporting these increases the awareness of risk and motivation to implement preventive action.

Thus, the importance of the device at one's fingertips that collects accurate information in real time is clear.

Near-falls were the most frequently reported event in both the smartphone and smartTV groups—over a third indoors (in the bathroom or toilet) and almost a third with preceding symptoms of dizziness (a prompt to seek medical attention). If not for the App, there was no other means for participants to report near-falls.

Fear of falling comprises psychosocial, physical and global function factors that leave individuals with a lasting concern about falling and causes them to avoid activities that they are physically capable of performing. Though common among people who have already suffered a fall, the fear of falling was most reported in the older cohort in this study (the smartTV users). The number of prior falls significantly affected the fear of falling. Users who had not suffered a prior fall at baseline reported less fear of falling than users who had fallen once and twice. A history of one fall was enough to create the fear of falling in the future and motivated participants to watch the exercise video. Self-reports of the fear of falling should be followed up. Digital technology may provide a neutral, impassive platform for older adults to share their fears in the form of an App such as this. Addressing these fears is potentially protective. Litwin et al. (20) showed that among individuals with severe restrictions in mobility, the absence of the fear of falling was associated with a subsequent fall. Thus, addressing fears, building confidence and removing the hazards implicated in a potential fall are key. Srygley et al. (7) go further; linking missteps to subsequent falls, i.e., missteps mark a decline in postural control leading to a fall. Self-reports of the fear of falling, a misstep or near-fall should prompt intervention that could prevent a fall.

Moreover, since it is difficult to determine what comes first, falls, near-falls or the fear of falls, it is important to report all types of fall event because all of these events contribute to the downward spiral in functionality and mobility that result in a fall. The use of a validated falls scale may help standardize the reporting of falls, with potential benefits for clinical decision-making. Data about fall events from older users of technology may increase the data available to better map risk and target intervention in the community.

The App was of most value for those most at risk (the smartTV group) and for those who reported “no-falls.” Participants who experienced more “good day”/no-fall events were more likely to be at risk of falls as they failed to engage in fall mitigating activity. This is borne out in our finding that positive (a good day) and negative (fall) reports prompted participants to watch the exercise video. Thus, both groups benefit from the habit forming behaviors promoted in the App, such as undertaking regular exercise (34). Exercising may have an important role in optimizing older adults' health and prevention of the next fall. Clearly, forcing oneself to acknowledge adverse events through self-reports of silent events is a stronger motivator for change and adherence to fall prevention interventions, including habit-forming long-term preventative measures.

In this research, fall prevention is about optimizing independence and managing risk. This requires the management of medical comorbidities through timely monitoring, an understanding of where hazards are, when risk exists and taking lasting action to improve health and mitigate risk before complications ensue.

At the final focus group with the users, we compared the App to other devices currently in use for fall prevention, such as smart watch sensors and an alarm pendant. It was concluded that the App is a “pre-alarm pendant” because its utility is *before* rather than during a fall event. In addition, the App has value in rehabilitation, confidence building and return to normal levels of mobility *after* a fall, as well as prevention of a further fall. The potential in facilitating older adults to exert control or responsibility over their own health is not to be underestimated. The self-management App, comprising a self-reporting and exercise promotion tool, constitutes healthy aging as a contextual phenomenon for older adults related to their surroundings, behaviors, habits, and positive and negative life-events enabling people to manage risk. This was in contrast to the alarm pendant which conveys a specific version of aging where older adults feel continually aware of risk and danger and the need for emergency assistance—associated with a greater perception of vulnerability than the means to mitigate the next fall that the App promotes. The facility for older adults to negotiate ideas and practices of risk and safety—the hallmark of the *aging in place* and healthy aging discourse—is important to them in their acceptance of dynamic and diverse technology.

### Limitations

We have no subsequent data on whether watching the video or performing the exercises prevented a fall in the months after the study concluded. Similarly, we have no follow up data on whether reporting silent events went on to prevent falls in subsequent months. We know that the video was watched to the end but do not know how well the exercises were performed by every individual. Participants in the final focus group reported that they enjoyed the video and did perform the exercises.

We would have liked to have introduced more health interventions based on information collected from the smartTV and smartphone users about their fears of falling, the hazards they perceived and the causes of the fall events they experienced. Removing these hazards and making the environment safer indoors are key in fall prevention. Our study groups reported the fear of falling on public buses.

With over 30% of people aged 65 years or older owning and using tablets, perhaps tablets are a better choice for App delivery, especially for the oldest users; certainly, the video is easier to watch on a device larger than a smartphone. Voice recognition may further increase acceptance and utility of digital health technology.

Apps such as these do record personal information. In many ways, the more information stored on the App the greater the value of the App and better tailored the intervention. Individuals are, however, entering personal health and social information, and issues of data privacy will increasingly be a focus of concern as health and social care come to rely on digital technology.

We hope to proceed with further research exploring other interventions, such as prompts to make appointments with a physician, upload images of hazards encountered in and outdoors which prompt intervention from App users, family members and caregivers. Fall prevention interventions include balance and gait exercises, eye tests and visual aids, addressing the effects and side effects of medication and polypharmacy, removing hazards and making environments safer when mobilizing (in the home or in public spaces), and addressing deteriorations in chronic conditions^6^.

## Conclusion

As access to healthcare presents an increasingly formidable challenge in an aging global population, this timely study adds to research into how information technologies may be designed for older adults and used to identify and mitigate risk, and promote healthy aging. Apps such as “Age-Techcare” will need to be further developed and interface with health and social care services to keep populations well, link them with services that monitor health and prevent adverse health events. Further development of applications that prompt diverse interventions to promote healthy aging are to be encouraged.

## Summary

### What Was Already Known on This Topic:

- The incidence of falls is poorly documented
- Silent fall events are frequently undocumented
- Exercise is an effective intervention in the prevention of falls
- No App nor mobile technology exists that links accurate documentation and the prevention of falls.

### What This Study Added to our Knowledge:

- Accurate reporting and documentation of falls is effective using this self-reporting App
- Older adults find an App such as this easy to use and helpful, especially those who have a fear of falling and a history of silent fall events (those at a high risk of falling)
- This App effectively links documentation, self-awareness and a protective intervention
- A self-management tool is valuable when based on a feedback loop which links accurate documentation and the prevention of falls.

## Data Availability Statement

All datasets generated for this study are included in the article/supplementary material.

## Ethics Statement

The studies involving human participants were reviewed and approved by the Ethics Committee of Hadassah Academic College in Jerusalem. The patients/participants provided their written informed consent to participate in this study.

## Author Contributions

KM conceived and designed all three phases of the research (including development of the App), collected and analyzed the data, and wrote the manuscript. SB wrote and revised the manuscript for important intellectual content. UL provided medical advice for study design.

## Conflict of Interest

The authors declare that the research was conducted in the absence of any commercial or financial relationships that could be construed as a potential conflict of interest.

## Footnotes

^1^WHO. Retrieved from: https://www.who.int/news-room/fact-sheets/detail/falls (accessed August 5, 2019).

^2^For more about fall risk factors see also the STEADI pocket. Available online at: https://www.cdc.gov/steadi/pdf/STEADI-PocketGuide-508.pdf (accessed March 18, 2020).

^3^The Israeli National Insurance funds. Available online at: https://www.btl.gov.il/English%20Homepage/Pages/default.aspx (accessed March 18, 2020).

^4^The American Jewish Joint Distribution Committee Available online at: https://www.jdc.org/ (accessed March 18, 2020).

^5^Uniper care technologies. Available online at: https://www.Unipercare.com/en/home (accessed March 18, 2020).

^6^The National Council on Aging. Available online at: https://www.ncoa.org/healthy-aging/falls-prevention/ (accessed March 2, 2020).

## References

[1] Rubenstein LZ. Falls in older people: epidemiology, risk factors and strategies for prevention. Age Aging. (2006) 35:ii37–ii41. 10.1093/ageing/afl084 16926202

[2] Ambrose AF Paul G Hausdorff JM. Risk factors for falls among older adults: a review of the literature. Maturitas. (2013) 75:51–61. 10.1016/j.maturitas.2013.02.009 23523272

[3] Boyd R Stevens JA. Falls and fear of falling: burden, beliefs and behaviours. Age Aging. (2009) 38:423–8. 10.1093/ageing/afp053 19420144

[4] Dunsky A. The effect of balance and coordination exercises on quality of life in older adults: a mini-review. Front Aging Neurosci. (2019) 11:318. 10.3389/fnagi.2019.00318 31803048 PMC6873344

[5] World Health Organization. Falls (2018). Retrieved from https://www.who.int/news-room/fact-sheets/detail/falls (accessed August 5, 2019).

[6] Florence CS Bergen G Atherly A Burns ER Stevens JA Drake C. Medical costs of fatal and nonfatal falls in older adults. J Am Geriatr Soc. (2018) 66:693–8. 10.1111/jgs.15304 29512120 PMC6089380

[7] Srygley JM Herman T Giladi N Hausdorff JM. Self-report of missteps in older adults: a valid proxy of fall risk? Arch Phys Med Rehab. (2009) 90:786–92. 10.1016/j.apmr.2008.11.007 19406298 PMC3180816

[8] Freiberger E De Vreede P. Falls recall—limitations of the most used inclusion criteria. Eur Rev Aging Phys Act. (2011) 8:1–4. 10.1007/s11556-011-0078-9

[9] Tinetti ME Kumar C. The patient who falls: It's always a trade-off. JAMA. (2010) 303:258–66. 10.1001/jama.2009.2024 20085954 PMC3740370

[10] Poss JW Hirdes JP. Very frequent fallers and future fall injury: continuous risk among community-dwelling home care recipients. J Aging Health. (2016) 28:587–99. 10.1177/0898264315599941 26270720

[11] Lamb SE McCabe C Becker C Fried LP Guralnik JM. The optimal sequence and selection of screening test items to predict fall risk in older disabled women: The Women's Health and Aging Study. J Gerontol A Biol Sci Med Sci. (2008) 63:1082–8. 10.1093/gerona/63.10.1082 18948559

[12] Panel on Prevention of Falls in Older Persons American Geriatrics Society and British Geriatrics Society. Summary of the Updated American Geriatrics Society/British Geriatrics Society clinical practice guideline for prevention of falls in older persons. J Am Geriatr Soc. (2011) 59:148–57. 10.1111/j.1532-5415.2010.03234.x 21226685

[13] Davalos-Bichara M Lin FR Carey JP Walston JD Fairman JE Schubert MC. Development and validation of a falls-grading scale. J Geriatr Phys Ther. (2013) 36:63–7. 10.1519/JPT.0b013e31825f6777 22810170 PMC3867809

[14] Wolf SL Barnhart HX Kutner NG McNeely E Coogler C Xu T. Reducing frailty and falls in older persons: an investigation of tai chi and computerized balance training. Atlanta FICSIT Group Frailty and Injuries: Cooperative Studies of Intervention Techniques. J Am Geriatr Soc. (1996) 44:489–97. 10.1111/j.1532-5415.1996.tb01432.x 8617895

[15] Teno J Kiel DP Mor V. Multiple stumbles: a risk factor for falls in community dwelling elderly. A prospective study. J Am Geriatr Soc. (1990) 38:1321–5. 10.1111/j.1532-5415.1990.tb03455.x 2254571

[16] Arnold CM Faulkner RA. The history of falls and the association of the Timed Up and Go Test to falls and near-falls in older adults with hip osteoarthritis. BMC Geriatr. (2007) 7:17. 10.1186/1471-2318-7-17 17610735 PMC1936991

[17] Zecevic AA Salmoni AW Speechley M Vandervoort AA. Defining a fall and reasons for falling: comparisons among the views of seniors, health care providers, and the research literature. Gerontologist. (2006) 46:367–76. 10.1093/geront/46.3.367 16731875

[18] Hauer K Lamb SE Jorstad EC Todd C Becker C. Systematic review of definitions and methods of measuring falls in randomised controlled fall prevention trials. Age Aging. (2006) 35:5–10. 10.1093/ageing/afi218 16364930

[19] Dickens J Jones M Johansen A. Falls definition–reliability of patients' own reports. Age Aging. (2006) 35:450–1. 10.1093/ageing/afl016 16641141

[20] Litwin H Erlich B Dunsky A. The complex association between fear of falling and mobility limitation in relation to late-life falls: a SHARE-based analysis. J Aging Health. (2018) 30:987–1008. 10.1177/0898264317704096 28553817 PMC6655432

[21] Friedman SM Munoz B West SK Rubin GS Fried LP. Falls and fear of falling: Which comes first? A longitudinal prediction model suggests strategies for primary and secondary prevention. J Am Geriatr Soc. (2002) 50:1329–35. 10.1046/j.1532-5415.2002.50352.x 12164987

[22] Greenberg. Assessment of Fear of Falling in Older Adults: The Falls Efficacy Scale International (FES-I). Hartford Institute for Geriatric Nursing; New York University Rory Meyers College of Nursing (2019). Retrieved from https://consultgeri.org/try-this/general-assessment/issue-29.pdf (accessed on July 27, 2020).

[23] Krishna S Boren SA Balas EA. Healthcare via cell phones: a systematic review. Telemed e-Health. (2009) 15:231–40. 10.1089/tmj.2008.0099 19382860

[24] Blake H. Mobile phone technology in chronic disease management. Nurs Stand. (2008) 23:43–6. 10.7748/ns.23.12.43.s56 19093357

[25] Glynn LG Murphy AW Smith SM Schroeder K Fahey T. Self-monitoring and other non-pharmacological interventions to improve the management of hypertension in primary care: a systematic review. Br J Gen Pract. (2010) 60:e476–88. 10.3399/bjgp10X544113 21144192 PMC2991764

[26] Morrissey EC Glynn LG Casey M Walsh JC Molloy GJ. New self-management technologies for the treatment of hypertension: general practitioners' perspectives. Fam Pract. (2017) 35:318–22. 10.1093/fampra/cmx100 29088438

[27] Bengtsson U Kjellgren K Höfer S Taft C Ring L. Developing an interactive mobile phone self-report system for self-management of hypertension. Part 2: Content validity and usability. Blood Pressure. (2014) 23:296–306. 10.3109/08037051.2014.901009 24786778 PMC4196575

[28] Morton K Dennison L May C. Using digital interventions for self-management of chronic physical health conditions: a meta-ethnography review of published studies. Patient Educ Couns. (2017) 100:616–35. 10.1016/j.pec.2016.10.019 28029572 PMC5380218

[29] Kwon S. (ed.). Gerontechnology: Research, Practice, and Principles in the Field of Technology and Aging. New York, NY: Springer (2017). 10.1891/9780826128898

[30] Jia F Lu Y Wajda B. Designing for technology acceptance in an aging society through multi-stakeholder collaboration. Procedia Manuf . (2015) 3535–42. 10.1016/j.promfg.2015.07.701

[31] Mannheim I Schwartz E Xi W Buttigieg S McDonnell-Naughton M Wouters E. Inclusion of older adults in the research and design of digital technology. Int. J Environ Res Public Health. (2019) 16:3718. 10.3390/ijerph16193718 31581632 PMC6801827

[32] Peine A Neven L. The co-constitution of ageing and technology – a model and agenda. Ageing Soc. (2020) 1–22. 10.1017/S0144686X20000641

[33] Peek ST Wouters EJ Van Hoof J Luijkx KG Boeije HR Vrijhoef HJ. Factors influencing acceptance of technology for aging in place: a systematic review. Int J Med Inform. (2014) 83:235–48. 10.1016/j.ijmedinf.2014.01.004 24529817

[34] Parry SW Steen N Galloway SR Kenny RA Bond J. Falls and confidence related quality of life outcome measures in an older British cohort. Postgrad Med J. (2001) 77:103–8. 10.1136/pmj.77.904.103 11161077 PMC1741890

